# Risk factors associated with dengue lethality: case-control study, Joinville, Brazil

**DOI:** 10.1590/0037-8682-0165-2025

**Published:** 2026-03-04

**Authors:** Romana Pedott Apel, Saulo Vicente Rocha, Dorceli Alves Lemes da Cruz, Juliana Antunes Safanelli, Helbert do Nascimento Lima

**Affiliations:** 1 Universidade da Região de Joinville, Departamento de Ciências Biológicas, Joinville, SC, Brasil.; 2 Secretaria Municipal de Saúde de Joinville, Joinville, SC, Brasil.; 3 Universidade da Região de Joinville, Programa de Pós-Graduação em Saúde e Meio Ambiente, Joinville, SC, Brasil.

**Keywords:** Dengue, Mortality, Case-control studies, Risk factors, Brazil, Fluid therapy, Warning signs

## Abstract

**Background:**

Dengue remains a major public health concern in Brazil, with approximately 6 million cases and over 6,000 deaths reported in 2024. Joinville, in southern Brazil, recorded one of the highest incidence rates in the country. Understanding the clinical and demographic factors associated with dengue-related mortality in this region is essential for developing effective interventions.

**Methods:**

We conducted a retrospective matched case-control study using data from the Sistema de Informação de Agravos de Notificação database, involving adult patients with dengue who presented with warning signs or severe disease and were treated within Joinville's public health system (Sistema Único de Saúde) between January 2021 and December 2024. Cases were defined as fatal outcomes, while controls were matched survivors. Univariate and multivariate logistic regression models were used to estimate mortality risk.

**Results:**

Among 454 patients, 116 (26%) were cases and 336 (74%) were controls. Lower mortality was associated with having ≥ 2 medical consultations (odds ratio [OR] = 0.20; 95% confidence interval [CI]: 0.10-0.51) and receiving intravenous hydration of ≥ 1 L (OR = 0.32; 95% CI: 0.14-0.72). Hypotension (OR = 3.06; 95% CI: 1.33-7.03), thrombocytopenia (OR = 2.47; 95% CI: 1.21-5.02), and lethargy or irritability (OR = 15.59; 95% CI: 6.50-37.38) were significantly associated with mortality.

**Conclusions:**

Early intravenous rehydration and repeated clinical evaluations were associated with reduced mortality. Close monitoring of patients with warning signs is crucial for improving outcomes in high-incidence settings.

## INTRODUCTION

Since 1983, dengue has been the official designation for the arthropod-borne disease in Brazil and is caused by a virus of the *Orthoflavivirus* genus within the *Flaviviridae* family^1^ ^,^ ^2^. Over the past 5 years, the number of cases has risen significantly, and dengue has re-emerged as a serious public health concern^3^. In 2024, approximately 6 million dengue cases were confirmed nationwide in Brazil, with approximately 6,000 resulting in death^4^ ^,^ ^5^. Joinville, the largest city in the state of Santa Catarina, reported one of the highest dengue incidence rates in the country^5^ ^,^ ^6^. However, few studies have examined the characteristics of fatal dengue cases among patients with clinically severe presentations. 

The etiological agent of dengue, *Orthoflavivirus dengue*, comprises four virologically distinct serotypes (DENV-1, DENV-2, DENV-3, and DENV-4), which are primarily transmitted in South America by the mosquito vector *Aedes aegypti* ^1^ ^,^ ^2^ ^,^ ^7^. The World Health Organization classifies the disease into three categories based on clinical signs and symptoms: classic dengue, dengue with warning signs, and severe dengue^7^ ^-^ ^9^. Epidemiological data from the Brazilian Ministry of Health indicate that in 2024, approximately 1.7% of confirmed cases in Brazil were classified as dengue with warning signs, whereas 0.14% were categorized as severe dengue. The overall case fatality rate was approximately 0.11%, with mortality reaching 5.8% among severe cases^5^. Although the pathophysiological mechanisms underlying high lethality are not yet fully understood, individuals with chronic comorbidities, children under 2 years of age, and adults over 65 years remain the most vulnerable groups.

Joinville, the third-largest city in southern Brazil, has an estimated population of approximately 616,000 inhabitants, according to the 2022 demographic census^10^. The city reported its first autochthonous dengue cases in 2019^11^. Since then, the municipality has experienced a rapid expansion of the disease, with incidence increasing from 1,453 cases per 100,000 inhabitants in 2020 to 12,249 cases per 100,000 in 2024^5^. In parallel, dengue-related deaths rose substantially, from five cases in 2021 to 83 in 2024, corresponding to a case fatality rate of 0.10% among confirmed cases^5^. The circulating serotypes identified in the region were DENV-1 and DENV-2, with a clear predominance of DENV-1^12^. 

Given the current epidemiological situation, characterized by widespread transmission and increasing lethality, a deeper understanding of the factors associated with fatal outcomes is essential. This study aims to identify and analyze the clinical and demographic risk factors associated with mortality among patients diagnosed with dengue who presented warning signs or severe clinical manifestations in Joinville, the Brazilian city with the highest disease incidence in 2024.

## METHODS

### Study Design, Setting, and Sampling

This retrospective, matched case-control study utilized data from the national dengue surveillance database, the *Sistema de Informação de Agravos de Notificação* (SINAN). All adult patients in Joinville with laboratory-confirmed dengue who presented warning signs or severe disease and were treated within the Brazilian unified public health system (*Sistema Único de Saúde, SUS*) between January 2021 and December 2024 were included in the study. 

### Data Sources

SINAN is a national registry managed by the Brazilian Ministry of Health to monitor and record notifiable diseases, including dengue^12^ ^,^ ^13^. In Joinville, the local Epidemiological Surveillance Team (EST) evaluates and registers all suspected and confirmed cases within SINAN, classifying them according to disease severity. The EST comprises trained professionals who adhere to the criteria set by the Ministry of Health for verifying and monitoring dengue cases^13^. The team confirms all suspected cases through review of medical records, laboratory results, and, when necessary, contact with patients or their families. Information on parenteral hydration prescribed during medical care and number of medical consultations was also collected through review of electronic medical records.

### Ethical Considerations

This study was approved by the Institutional Research Ethics Committee (CAEE 83327624.8.0000.5366). Due to the retrospective and observational study design, the requirement for informed consent was waived.

### Case and Control Definition

Dengue cases were confirmed using laboratory criteria, including reverse transcription polymerase chain reaction, nonstructural protein 1 antigen testing, or serology, together with clinical and epidemiological assessment conducted by healthcare professionals from SUS. All patients who died of dengue were classified as cases. The control group consisted of patients with dengue with warning signs or severe dengue who survived. For each case, three controls were randomly selected from the same SINAN registry using frequency matching by sex and age (5-year age intervals). The sample size was defined by the total number of eligible cases available during the study period (January 2021 to December 2024), including all individuals in the SINAN database who met the inclusion criteria for both cases and controls.

### Variables Considered

The analyzed variables included sociodemographic characteristics (age, sex, race/skin color, and years of education), clinical history (number of medical consultations and interval from symptom onset to first consultation), and laboratory parameters (serum creatinine and urea levels measured during the first or second emergency care visit). Comorbidities documented in both the SINAN registry and emergency care medical records were considered, including diabetes, hypertension, heart failure, stroke, and cancer. The administration of at least 1 L of intravenous hydration during any emergency care visit was also recorded; this volume of fluid replacement was based on the estimated initial hydration recommendation for a 70-kg individual with severe dengue (10 mL/kg in the first hour)^13^. Furthermore, warning signs-including hypotension, thrombocytopenia, vomiting, bleeding, hemoconcentration, abdominal pain, lethargy or irritability, hepatomegaly, and fluid accumulation-were extracted from SINAN records.

### Statistical analysis

Categorical variables were described as frequencies and percentages, whereas continuous variables were summarized using means and standard deviations. Categorical variables were compared between cases and controls using the chi-square test, and numerical variables were analyzed using the Mann-Whitney U test. Univariate and multivariate analyses were performed using unconditional logistic regression, as controls were frequency matched by sex and age group rather than individually paired; therefore, cases and controls were treated as independent samples when assessing associations between variables and mortality^14^. Variables were initially selected for inclusion in the multivariate model based on clinical relevance, prior evidence, and a p-value < 0.10 in univariate analysis. The final model was refined through manual backward elimination, retaining clinically meaningful variables (such as sex and age) regardless of statistical significance. All analyses were conducted using STATA/IC version 15.1 (StataCorp LLC, College Station, TX, USA).

## RESULTS

**FIGURE 1:**
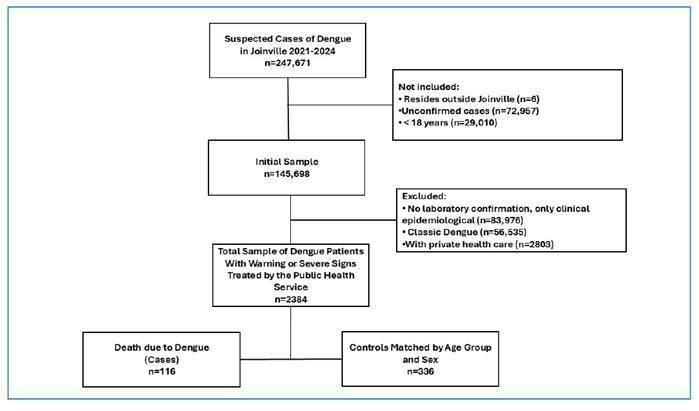
Flowchart of the sample.

From an initial sample of 145,698 patients diagnosed with dengue, 2384 were classified as having dengue with warning signs or severe dengue and were treated within the SUS. Among these, 116 individuals (2.0%) died, as shown in the participant selection flowchart (Figure 1). The final analytical sample comprised 454 individuals, including 116 cases (26%; deaths) and 336 controls (74%; survivors). Compared with the control group, patients who died had a lower educational level (58.7% with ≥ 5 years of education vs. 83.1%; p < 0.001), a lower median number of medical consultations, and lower hematocrit levels. Additionally, they exhibited higher urea levels and a greater prevalence of comorbidities, including diabetes mellitus, systemic arterial hypertension, heart failure, stroke, and cancer. Regarding clinical warning signs, a significantly higher frequency of hypotension, thrombocytopenia, hemoconcentration, lethargy or irritability, and fluid accumulation was observed among fatal cases (p < 0.05). Table 1 presents the clinical and laboratory characteristics of both groups.

**TABLE 1:** Demographic and clinical characteristics of patients who survived or did not after severe Dengue or Dengue with warning signs, Joinville, Brazil (2021-2024).

**Variable**	Control/Survived n=336 (74.0%)		Case/Died n=118 (26.0%)		p-value
	Total or Median	% or IQR	Total or Median	% or IQR	
**Age**	52.0	30.5/72.1	52.9	32.6/73.6	0.484
18-36 years	115	34.2%	36	30.5%	
37-65 years	111	33.0%	40	33.9%	0.743
>65 years	110	32.7%	42	35.6%	
**Sex**					0.976
Female	176	52.4%	62	52.5%	
Male	160	47.6%	56	47.5%	
**Skin color**					0.477
White	309	92.0%	106	89.8%	
Non-white	27	8.0%	12	10.2%	
**Years of education,** n=337					
0-4 years	44	16.8%	31	41.3%	<0.001
5-8 years	128	48.8%	29	38.7%	
≥9 years	90	34.3%	15	20.0%	
**Number of medical consultations**	2	1/2	1	1/2	<0.001
<2	141	42.0%	88	74.6%	<0.001
≥2	195	58.0%	30	25.4%	
**Time from symptom onset to first consultation** (days)	2	1/3	2	1/4	0.8113
<2 days	135	40.2%	49	41.5%	0.798
≥2 days	201	59.8%	69	58.5%	
**Creatinine,** mg/dl; n=154	0.8	0.7/1.1	0.9	0.7/1.4	0.112
**Urea,** mg/dl; n=137	30	20/44	49	36/73	<0.001
**Pre-existing comorbidities** (yes)					
Diabetes	54	16.1%	33	28.0%	0.005
Hypertension	138	41.1%	71	60.2%	<0.001
Heart failure	9	2.7%	9	7.6%	0.018
Stroke	11	3.3%	15	12.7%	<0.001
Cancer	4	1.2%	8	6.8%	0.003
**Received** ≥**1 liter of IV saline** (yes)	171	(50.9%)	38	32.2%	<0.001
**Warning signs** (yes)					
Hypotension	56	(16.7%)	67	(57.3%)	<0.001
Thrombocytopenia	140	(41.7%)	81	(69.2%)	<0.001
Vomiting	37	(11.0%)	20	(17.2%)	0.081
Bleeding	64	(19.0%)	30	(25.9%)	0.119
Hemoconcentration	8	(2.4%)	9	(7.8%)	0.009
Abdominal pain	141	(42.0%)	42	(35.9%)	0.249
Lethargy/irritability	19	(5.6%)	64	(55.2%)	<0.001
Hepatomegaly	2	(0.6%)	1	(0.9%)	0.760
Fluid accumulation	5	(1.5%)	24	(20.7%)	<0.001

**IQR:** interquartile range (percentile 25^th^/percentile 75^th^); **IV:** intravenous.

Table 2 presents the univariate associations between clinical variables and mortality risk. Patients who died were less likely to have had two or more medical consultations (odds ratio [OR] = 0.25; 95% confidence interval [CI]: 0.15-0.39) or to have received at least 1 L of intravenous saline hydration (OR = 0.46; 95% CI: 0.29-0.71) compared with those who survived. In contrast, previous diagnoses of diabetes mellitus, systemic arterial hypertension, and stroke were more frequent among fatal cases. Similarly, warning signs such as hypotension, thrombocytopenia, hemoconcentration, and lethargy or irritability were significantly more common among patients who died in the univariate analysis. 

**TABLE 2:** Univariate and multivariate analysis of variables associated with a higher risk of death due to Dengue.

Variable	Category	Univariate			Multivariate		
		OR	95% CI	p-value	OR	95% CI	p-value
**Age**	per year increase	1.00	0.99-1.01	0.455	1.01	0.99-1.03	0.135
**Sex**	Female	1.00	Reference		1.00	Reference	
	Male	0.99	0.65-1.51	0.976	0.92	0.45-1.87	0.815
**Years of education**	0-4 years	1.00	Reference		1.00	Reference	
	5-8 years	0.32	0.17-0.59	<0.001	0.48	0.20-1.12	0.090
	≥9 years	0.23	0.11-0.48	<0.001	0.63	0.24-1.67	0.356
**More than one consultation**	No	1.00	Reference		1.00	Reference	
	Yes	0.25	0.15-0.39	<0.001	0.22	0.10-0.51	<0.001
**Diabetes**	No	1.00	Reference				
	Yes	2.03	1.23-3.33	0.005			
**Hypertension**	No	1.00	Reference				
	Yes	2.17	1.41-3.32	<0.001			
**Previous stroke**	No	1.00	Reference		1.00	Reference	
	Yes	4.30	1.92-9.66	<0.001	3.83	0.89-16.54	0.072
**Hypotension**	No	1.00	Reference		1.00	Reference	
	Yes	6.70	4.20-10.67	<0.001	3.06	1.33-7.03	0.008
**Thrombocytopenia**	No	1.00	Reference		1.00	Reference	
	Yes	3.15	2.01-4.93	<0.001	2.47	1.21-5.02	0.012
**Vomiting**	No	1.00	Reference				
	Yes	1.68	0.93-3.04	0.084			
**Hemoconcentration**	No	1.00	Reference				
	Yes	3.45	1.30-9.16	0.013			
**Lethargy/Irritability**	No	1.00	Reference		1.00	Reference	
	Yes	20.53	11.38-37.05	<0.001	15.59	6.50-37.38	<0.001
**Received** ≥**1L IV fluids**	No	1.00	Reference		1.00	Reference	
	Yes	0.46	0.29-0.71	0.001	0.32	0.14-0.72	0.006

**IV:** intravenous.

After adjusting for potential confounding variables, patients who died remained less likely to have had two or more medical consultations (OR = 0.22; 95% CI: 0.10-0.51) or to have received at least 1 L of intravenous saline hydration (OR = 0.32; 95% CI: 0.14-0.72). Conversely, warning signs such as hypotension (OR = 3.06; 95% CI: 1.33-7.04), thrombocytopenia (OR = 2.47; 95% CI: 1.21-5.02), and lethargy or irritability (OR = 15.59; 95% CI: 6.50-37.88) were independently associated with mortality in the multivariate analysis.

## DISCUSSION

This case-control study demonstrated that patients with severe dengue or clinical warning signs, particularly hypotension, thrombocytopenia, or lethargy, had a significantly higher risk of death. These findings directly address the study’s objective of identifying independent clinical and demographic predictors of dengue-related mortality. Conversely, the analysis revealed that both intravenous fluid therapy and multiple clinical consultations were independently associated with a lower likelihood of death, highlighting the critical role of timely diagnosis, adequate supportive care, and ongoing clinical monitoring in preventing fatal outcomes.

Although not the primary focus of this study, it is important to emphasize that the case fatality rates for confirmed and suspected dengue cases in Joinville in 2024 (0.1 and 2.7 per 100,000 cases, respectively) were comparable to the national averages for the same year (0.1 and 5.9 per 100,000 cases, respectively)^5^. 

Regarding demographic factors, neither age nor sex was significantly associated with mortality, likely due to the frequency-matching process used to select controls, which may have limited the observable effects of these variables. Although advanced age (> 60 years) has been linked to increased dengue-related fatality^2^ ^,^ ^15^ ^-^ ^19^, the association with higher mortality has not been consistently observed^20^ ^-^ ^24^. Similarly, previous research has not demonstrated a consistent relationship between sex and dengue mortality^2^ ^,^ ^15^ ^,^ ^25^. Conversely, lower educational attainment and the presence of comorbidities were more prevalent among patients who died in this study. Lower educational attainment has been associated with increased dengue mortality, reflecting the influence of social determinants on disease outcomes. Individuals with less education may have reduced health literacy, delayed recognition of warning signs, and limited access to timely healthcare, all of which can contribute to a worse prognosis. Studies conducted in Brazil and Asia have reported higher case fatality rates among populations with lower education or residing in socioeconomically deprived areas, reinforcing the link between educational disparities and dengue lethality^26^ ^-^ ^29^.

The presence of preexisting comorbidities has been linked to a heightened risk of death among patients with dengue^30^. A case series study based on health records from Brazil, Mexico, and Colombia revealed an increased risk of death among patients with comorbidities such as ischemic heart disease (OR = 23; 95% CI: 6.6-79.6), kidney disease (OR = 8.3; 95% CI: 4.8-14.2), and pulmonary diseases (OR = 11.6; 95% CI: 7.4-18.2)^31^. 

Similarly, a retrospective cohort study involving hospitalized patients with confirmed hemorrhagic dengue from 5,983 hospitals in Brazil found that comorbidities, such as kidney disease (relative mortality risk [RMR] = 107, 95% CI: 30-383, p < 0.001) and diabetes (RMR = 173, 95% CI: 32-950, p < 0.01), were associated with a significantly increased risk of death^32^. An ecological study conducted in Joinville identified that neighborhoods with low household income and inadequate sewage coverage experienced higher dengue incidence between 2020 and 2021, reinforcing the role of social vulnerability in the dynamics of the disease within the municipality^33^. Lower educational attainment has similarly been linked to higher dengue mortality, particularly among young individuals, potentially increasing the risk of fatality by up to 70%^34^.

Clinical warning signs and laboratory abnormalities are consistently associated with poorer outcomes in dengue^2^. A systematic review of 17 studies found that thrombocytopenia in patients with dengue was associated with a 2- to 5-fold increased risk of death^35^. Thrombocytopenia is one of the most frequently reported hematological abnormalities in severe dengue, and serves as a critical marker of disease severity and increased mortality risk^36^. When accompanied by elevated hematocrit levels, thrombocytopenia is the most frequently reported hematologic abnormality in severe dengue cases and is strongly linked to increased capillary permeability and plasma leakage, which are key factors in the progression to shock^37^. In this study, both thrombocytopenia and hemoconcentration were more prevalent among fatal cases. Acute kidney injury is another significant clinical complication of dengue and has been consistently identified as an independent predictor of mortality in adult patients across several studies^20^ ^,^ ^21^ ^,^ ^23^, underscoring its critical role in disease severity and fatal outcomes. However, in this study, kidney function was not routinely assessed, limiting its inclusion in the analysis.

Volume replacement has been strongly advocated as an early intervention to reduce dengue-related mortality^2^ ^,^ ^9^ ^,^ ^38^. Accordingly, guidelines from the Brazilian Ministry of Health recommend administering 10 mL/kg of normal saline for patients in Group C (those with warning signs) and 20 mL/kg within 20 minutes for patients in Group D (severe dengue)^9^. In this study, patients who died received intravenous hydration less frequently, and only 50% of survivors received the minimum recommended volume. After adjusting for other variables, patients who received 1 L or more of intravenous saline had a lower likelihood of death. A previous review demonstrated that early volume replacement during the critical phase of dengue reduces mortality^39^, as adequate hydration decreases the risk of dengue-related complications^39^ ^,^ ^40^. However, evidence from a large cohort in China suggests that intravenous fluid therapy in patients with dengue without shock does not reduce the risk of progression to severe disease and may increase the likelihood of fluid overload and pulmonary complications^24^. These findings underscore the importance of exercising careful clinical judgment when initiating fluid replacement, balancing the benefits of early rehydration with the potential risks of overhydration.

Fluid replacement remains the cornerstone of management for patients with severe dengue or warning signs. Early and adequate intravenous hydration has been shown to reduce mortality by preventing hypovolemic shock resulting from plasma leakage^41^. Clinical and observational studies have consistently demonstrated that underhydration and delayed fluid replacement are associated with poorer outcomes in hospitalized patients^20^ ^,^ ^21^. However, recent evidence indicates that excessive fluid administration can be harmful. A large cohort study from China reported that high intravenous fluid volumes in patients without shock did not prevent progression to severe dengue and were associated with fluid overload and pulmonary complications^24^. Similarly, a study from Sri Lanka found that fluid volumes exceeding recommended thresholds were linked to an increased risk of severe plasma leakage^42^. These findings underscore the importance of early yet judicious fluid management to strike a balance between adequate resuscitation and potential risks of overhydration.

In this study, a greater number of medical consultations during dengue was associated with a reduced risk of mortality. This finding highlights the importance of enhanced clinical monitoring and access to healthcare services, which are critical for the early detection of complications and timely therapeutic interventions. Continuous monitoring of clinical and laboratory parameters is essential for promptly identifying complications and providing timely interventions, thereby reducing the risk of progression to severe dengue and shock^36^ ^,^ ^43^. Rapid access to well-equipped healthcare facilities is also crucial for lowering mortality by preventing delays in fluid resuscitation and intensive care^19^ ^,^ ^44^. Efficient triage and prioritization of critically ill patients further improve clinical outcomes and optimize healthcare system capacity^45^. Although the specific management during medical consultations was not assessed, and no differences were observed in the time to first consultation after symptom onset in the studied sample, the number of consultations may reflect a higher level of care received by patients who survived. 

Other variables independently associated with an increased risk of death included thrombocytopenia, hemoconcentration, and lethargy or irritability. Altered consciousness, in particular, has been strongly associated with mortality. A study involving patients with dengue in Taiwan reported an OR of 7.06 (95% CI: 2.19-22.73)^46^. Similarly, a retrospective cohort study by Sauceda-Acosta et al.^19^, conducted in Honduras between 2016 and 2022, identified lethargy as a significant risk factor for dengue-related mortality (OR: 1.80; 95% CI: 1.25-2.59), suggesting an association with cerebral hypoperfusion, systemic inflammation, or imminent shock. These findings underscore the need for close monitoring and early intervention in patients presenting with these warning signs. 

This study has several significant limitations. First, the retrospective nature of the medical records may have introduced information bias due to underreporting or data entry errors, potentially leading to underestimation or overestimation of the observed associations. Second, the lack of detailed information on hospital management limited our ability to assess the quality and consistency of clinical interventions, which could have influenced outcomes. Third, the absence of patient body weights in most medical records prevented individualized calculation of fluid replacement targets. Consequently, we relied on an approximate reference value based on the recommended first-hour infusion volume for a 70-kg adult (10 mL/kg), which may have introduced measurement bias and did not necessarily reflect the optimal fluid volume for each patient. Despite these limitations, the large sample size and use of controls from the same population strengthen the internal validity and relevance of the findings regarding dengue mortality in Joinville. This study contributes to a deeper understanding of severe dengue cases that progress to fatal outcomes. In addition to public health measures to combat dengue, such as rapid investigation of suspected cases and implementation of dengue vaccination^4^, improved clinical care for individuals affected by the disease remains critically needed. Our findings support the importance of ensuring timely access to healthcare, continuous patient monitoring, and adequate intravenous hydration for the most severe cases, which can be challenging during peak overcrowding in emergency departments throughout disease progression. 

## CONCLUSION

These findings emphasize the critical importance of timely intravenous rehydration and close monitoring of high-risk patients with dengue or those exhibiting warning signs to prevent severe outcomes. Future research should prioritize the implementation of effective re-evaluation protocols and enhanced monitoring strategies for severe dengue cases. Furthermore, strategies to improve adherence to recommended intravenous hydration as a preventive measure against mortality warrant further investigation. Integrating these findings into clinical practice, continuous patient evaluation, and the development of precise treatment protocols has the potential to significantly improve patient outcomes and reduce mortality rates in high-risk populations.

## Data Availability

Research data is only available upon request.
